# On the Hunt for Next-Generation Antimicrobial Agents: An Online Symposium Organized Jointly by the French Society for Medicinal Chemistry (Société de Chimie Thérapeutique) and the French Microbiology Society (Société Française de Microbiologie) on 9–10 December 2021

**DOI:** 10.3390/ph15040388

**Published:** 2022-03-23

**Authors:** Kevin Antraygues, Nina Compagne, Francesca Ruggieri, Kamel Djaout, Zainab Edoo, Maxime Eveque, Léo Faïon, Bruna Gioia, Salia Tangara, Anais Vieira Da Cruz, Baptiste Villemagne, Marion Flipo, Alain Baulard, Nicolas Willand

**Affiliations:** 1 University of Lille, Inserm, Institut Pasteur de Lille, U1177—Drugs and Molecules for Living Systems, F-59000 Lille, France; kevin.antraygues@univ-lille.fr (K.A.); nina.compagne.etu@univ-lille.fr (N.C.); francesca.ruggieri@univ-lille.fr (F.R.); maxime.eveque@univ-lille.fr (M.E.); leo.faion@univ-lille.fr (L.F.); bruna.gioia@univ-lille.fr (B.G.); salia.tangara@univ-lille.fr (S.T.); anais.vieira-da-cruz@inserm.fr (A.V.D.C.); baptiste.villemagne@univ-lille.fr (B.V.); marion.flipo@univ-lille.fr (M.F.); 2 University of Lille, CNRS, Inserm, CHU Lille, Institut Pasteur Lille, U1019—UMR 9017—CIIL—Center for Infection and Immunity of Lille, F-59000 Lille, France; kamel.djaout@ibl.cnrs.fr (K.D.); zainab.edoo@inserm.fr (Z.E.)

**Keywords:** antimicrobial resistance, drug discovery, microbiology

## Abstract

The restrictions posed by the COVID-19 pandemic obliged the French Society for Medicinal Chemistry (Société de chimie thérapeutique) and the French Microbiology Society (Société Française de Microbiologie) to organize their joint autumn symposium (entitled “On the hunt for next-generation antimicrobial agents”) online on 9–10 December 2021. The meeting attracted more than 200 researchers from France and abroad with interests in drug discovery, antimicrobial resistance, medicinal chemistry, and related disciplines. This review summarizes the 13 invited keynote lectures. The symposium generated high-level scientific dialogue on the most recent advances in combating antimicrobial resistance. The University of Lille, the Institut Pasteur de Lille, the journal Pharmaceuticals, Oxeltis, and INCATE, sponsored the event.

## 1. Aim and Scope of the Meeting

The rapid emergence of antimicrobial resistance (AMR) in bacteria is one of the most pressing global health threats. Despite the significance of this phenomenon, a large proportion of the general population underestimates its potential impact. The recent COVID-19 pandemic has intensified the use of antibiotics (often without clinical justification), which is expected to accentuate the AMR problem. More than 700,000 deaths per year worldwide (including 33,000 in Europe) are attributable to drug-resistant pathogens; this results in a substantial clinical and financial burden on healthcare providers, clinicians, patients, and carers. If we do not strengthen our efforts to stem the tide of AMR, antibiotic resistant infections might cause as many as 10 million deaths a year worldwide by 2050 [1].

Fortunately, many academic groups and biotech companies as well as a few large international pharmaceutical companies have chosen to take up the exciting challenge of replenishing the antibiotic development pipeline with innovative molecules. Experts in medicinal chemistry, drug design, biology, and microbiology presented their latest scientific advances over two half-days on 9–10 December 2021, as part of an online symposium organized by the French Society for Medicinal Chemistry (Société de Chimie Thérapeutique, SCT, www.sct-asso.fr, accessed on 17 March 2022) and the French Microbiology Society (Société Française de Microbiologie, SFM, www.sfm-microbiologie.org, accessed on 17 March 2022).

The SCT is a learned society founded in 1966. Its missions include the promotion of knowledge in key areas of pharmaceutical research and development, and particularly with regard to the discovery and validation of biological targets of therapeutic interest, screening, drug design, the optimization and selection of drug candidates, chemical biology, medicinal chemistry, pharmacokinetics (PK), metabolism, drug absorption, distribution, metabolism, excretion, and toxicity (ADME-T). The society organizes an annual international conference (Rencontres Internationales de Chimie Thérapeutique) covering the aspects mentioned above, a Young Researchers’ Meeting, and thematic workshops (spring and autumn meetings). The SCT awards a number of prizes to encourage research and underpin commitment to the field of medicinal chemistry. The society is a member of the French Federation of Chemical Sciences and the European Federation for Medicinal Chemistry. 

The SFM is a learned society founded in 1937. Its mission is to promote all fields of microbiology in French-speaking countries: human and veterinary medical microbiology, basic and general microbiology, food microbiology, environmental microbiology, biotechnology, virology, mycology, and biosafety. To this end, the society organizes or supports congresses and publishes a number of journals and books.

The online symposium opened with a few introductory words from the SCT president Professor Rebecca Deprez and SFM vice-president Professor Jean-Louis Herrmann.

## 2. Opening Lecture

Florence Séjourné, CEO at Da Volterra and President of BEAM Alliance provided the opening lecture: “Innovation in AMR: unlocking the late gates.” 

In addition to her role as CEO at Da Volterra (a late-stage French biopharmaceutical company developing innovative products to protect cancer patients from the consequences of antibiotic-induced intestinal dysbiosis), Florence Séjourné founded (in 2016) and currently chairs the BEAM Alliance (“Biotech companies from Europe innovating in AntiMicrobial resistance research”, a group of 70 European biotech companies developing innovative solutions for beating AMR). Prior to joining Da Volterra in 2008, Florence co-founded the French biotech company, GENFIT.

Her lecture began with a presentation on the BEAM Alliance and its activities, notably its role as a single voice for start-ups working on AMR and its development of policies and incentives in antimicrobial R&D in the context of the European Union’s “One Health” program. The BEAM Alliance has positioned itself as the key industry stakeholder for innovative AMR product development in Europe. In 2021, the Alliance had 51 full members, 3 international members, and 13 associate members. Florence Séjourné reminded the audience that no new classes of antibiotic (and especially those targeting Gram-negative bacteria) had been discovered since the 1980s. She stated that the present economic model for the commercialization and use of antibiotics is no longer appropriate and even scares off investors, since the costs of antibiotic R&D, production, and sales are generally higher than the potential revenues. In this context, big pharma and start-ups are all struggling to develop their assets. Therefore, push-and-pull incentives are needed to de-risk R&D and to secure the development portfolios. Today’s clinical pipeline of antibiotics will not be enough to counterbalance the emergence and spread of AMR. Nevertheless, preclinical drug development is currently very dynamic, with 292 antimicrobial compounds on the World Health Organization’s (WHO) priority list [2]. Nevertheless, innovation in the fight against Gram-negative bacteria must be continuously fuelled, in order to compensate for the inherent attrition of molecules during clinical development and ensure that enough pathogen-specific strategies emerge.

## 3. Keynote Lectures on Thursday, 9 December

### 3.1. Session 1: The Hunt for Active Natural Compounds

#### 3.1.1. Corramycin: A Novel Class of Natural Antibacterials from Myxobacteria, Cédric Couturier, Ph.D. (Evotec ID, Lyon, France)

Dr. Cédric Couturier obtained his Ph.D. in organic chemistry in Professor Jieping Zhu’s group at ICSN (Gif-sur-Yvette, France). He started his career as a medicinal chemist in Sanofi’s exploratory research department in 2005, with a focus on metabolism. He next joined the infectious disease group and worked on Gram-positive infections, tuberculosis, and malaria. In 2018, he joined Evotec as a group/project leader in the infectious disease department. His research interests include the design and synthesis of new Gram-negative antibiotics from natural products, as well as target-based approaches.

Natural products and their derivatives have played (and will continue to play) a key role in drug discovery. They account for a significant proportion of the marketed drugs currently used to treat a variety of human diseases, such as cancer, diabetes, and infectious diseases [3]. Despite the natural products’ major contributions as antibiotics, AMR (particularly the emergence of multidrug-resistant bacteria) still constitutes a global health problem [4,5]. Finding novel antibiotics that are active against resistant bacteria makes the task even more challenging.

**Figure 1:**
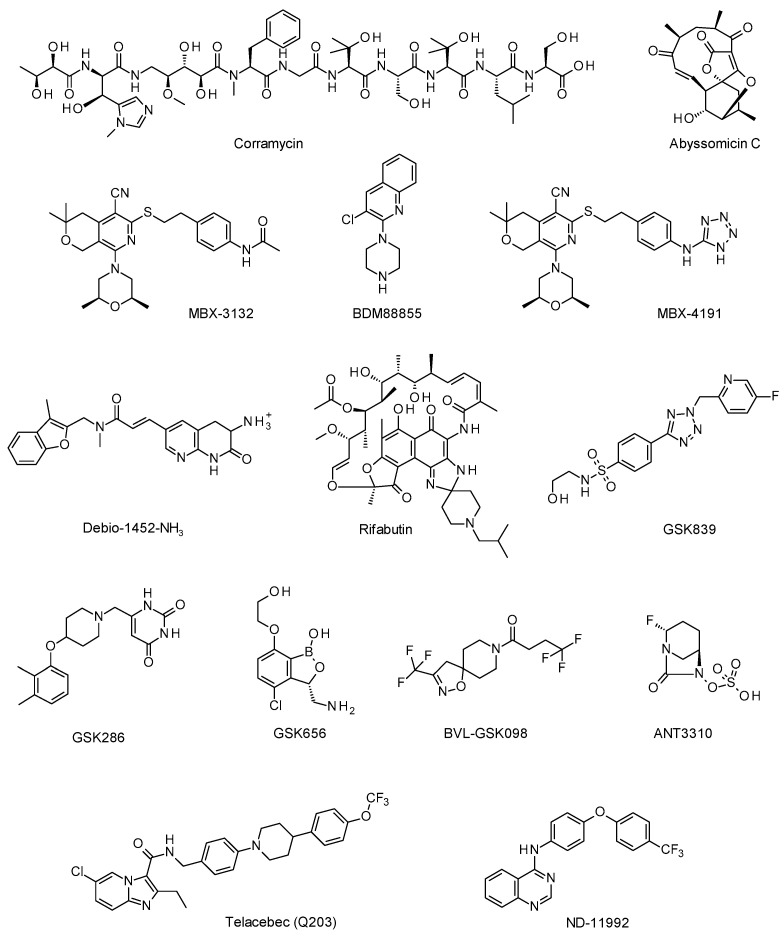
Chemical structures of compounds highlighted during the online symposium.

In this context, researchers at Sanofi used an activity-guided approach to isolate corramycin (Figure 1) from a culture of the myxobacterium Corallococcus coralloides. The compound’s structure was determined by combining NMR with total synthesis. Corramycin is a peptide compound with a novel scaffold containing eight α-amino acids (including the previously unknown histidine analogue δ-*N*-methyl-β-hydroxy histidine) and an unusual sugar moiety. The compound displayed a moderate level of activity (minimum inhibitory concentrations (MICs): 4 to 64 µg/mL) against several multidrug-resistant, Gram-negative bacteria, including *E. coli* ATCC 25922, *K. pneumoniae* 13883, and *A. baumannii* ATCC 1906, but was not active against Gram-positive bacteria. Corramycin exhibited good physicochemical and ADME properties, poor PK parameters, and remarkable in vivo efficacy in a model of *E. coli* septicaemia, thus making it a very attractive starting point for a lead optimization program.

Multiparameter lead optimization was initiated by Sanofi and Evotec and led to the synthesis of more than 800 corramycin analogues. Of these, the derivative “corramycin 3” was found to be at least 300 times more potent than native corramycin against *E. coli* ATCC 25922, *K. pneumoniae* 13883, and *A. baumannii* ATCC 1906 (MICs: from 0.015 to 0.031 µg/mL). It showed good ADME-PK properties and reasonable activity against *E. coli* and *K. pneumoniae* in vivo. Overall, “corramycin 3” is a promising candidate for the development of a new series of antibiotics against Gram-negative multidrug-resistant bacteria. Unexpectedly, corramycin inhibited gyrases very weakly, which suggests that the compound’s mode of action against Gram-negative bacteria is novel and has yet to be discovered.

#### 3.1.2. Antibiotic Discovery in the Abyss, Paul Race, Ph.D., Professor (University of Bristol, Bristol, United Kingdom)

Dr. Paul Race is Professor of Biological Chemistry at the University of Bristol. He is a founding Director of the Bristol BioDesign Institute (BBI) and is the Bristol lead for the EPSRC-funded UK Innovation and Knowledge Centre in Synthetic Biology (SynbiCITE). From 2014–2018, he served as Co-Director of the BBSRC/EPSRC-funded Bristol Centre for Synthetic Biology Research (BrisSynBio). His research focuses on the discovery of antimicrobial natural products and the development of functionally optimized “non-natural” derivatives of these important molecules.

The ocean is a continuing mystery to us, especially considering that less than 20% of its depth has been explored. In the last few years, Professor Paul Race’s research group has been exploring a small fraction of this hidden world. Their research is focused on two main questions: (i) “how does biology do chemistry?”, and (ii) “how can we exploit this knowledge for purposeful applications ?”. Professor Race presented three major aspects of his work, all of which have the abyss as a common denominator.

Discovery: One would expect the conditions encountered in the ocean’s abyss-extreme pressure, low temperature, and minimal light exposure to be inhospitable for life. However, some living things (such as sponges) have evolved to survive in this extreme environment. In general, these marine sponges house a microbiota that is always subjected to the extreme conditions of the deep sea. This “hostile” environment necessitates the development of metabolic innovations, novel biosynthetic capabilities, and thus the production of natural products that have never been tested in the clinic. This is why Professor Race’s group established the Bristol Sponge Microbiome Collection (BISECT) in 2020. BISECT is a unique repository of deep-sea bacteria and associated metabolites isolated from the microbiota co-existing with marine sponges in essentially unexplored regions of the mid-Atlantic Ocean. In an initial study focused on antibiotic discovery, the group found that 43 (6.2%) of the 699 isolated strains had antibacterial activity [6]. In subsequent work, the group isolated a new species of Micromonospora (“28ISP2-46T”) and studied its capacity to produce a diverse set of natural products. As a result, kosinostatin and isoquinocycline B were isolated from the bacterium’s fermentation broths and were found to be effective against the Gram-positive *S. aureus* SH1000 and the Gram-negative *K. pneumoniae* ATCC 10,031 and *A. baumannii* ATCC 17978 [7].

Biocatalysis: In the last few years, Professor Race’s group has investigated the biosynthesis of abyssomicin C, a spirotetronate antibiotic first isolated from the marine actinomycete Verrucosispora maris AB-18-032. This natural product is a potent inhibitor of bacterial folate metabolism and is effective against *Mycobacterium tuberculosis* and multidrug-resistant *S. aureus* [8]. In two recent publications, the group reported the structural and functional characterization of two enzymes involved in abyssomicin C (Figure 1) biosynthesis: the acetate lyase AbyA5 (able to catalyze an acetate-elimination reaction) [9], and the cyclase/Diels–Alderase AbyU (able to catalyze a [4 + 2] cycloaddition reaction) [10]. 

Applications: The group has demonstrated the existence of natural enzymes capable of catalyzing useful chemical reactions (such as a formal Diels–Alder cycloaddition) and able to accept non-natural substrates. This discovery paves the way for engineering these promising enzymes and/or exploiting them as biocatalysts for the synthesis of novel bioactive compounds.

### 3.2. Session 2: Blocking the Exit: Efflux Pump Inhibitors

#### 3.2.1. Multidrug Efflux Pumps: Approaches on How to Get Insight in Their Structure and Function, Klaas Martinus Pos, Ph.D., Professor (Goethe University, Frankfurt, Germany)

Dr. Klaas Martinus Pos is Professor of Membrane Transport Machineries at the Institute of Biochemistry, Goethe University Frankfurt, Germany. Using X-ray crystallography and cryo-electron microscopy, his research focuses on the molecular basis of drug multi-specificity, energy transduction, and the inhibition of bacterial multidrug efflux pumps.

Efflux pumps are transmembrane proteins that transport cytotoxic substances out of the cell. There are six (super)families of multidrug efflux pumps; the present seminar looked at a Gram-negative bacterial member of the Resistance Nodulation cell-Division (RND) superfamily of proton/drug antiporters, AcrAB-TolC, that catalyze the efflux of several classes of antibiotics across the outer membrane [11]. It is composed of outer membrane channel TolC and the RND-inner membrane transporter AcrB; both are connected by the tunnel-forming periplasmic adaptor protein AcrA. Each protomer of homotrimeric AcrB can adopt a different conformation: L (loose), T (tight), or O (open), representing consecutive states of a proposed drug transport cycle [12]. The putative drug transport mechanism through each protomer is based on the functional rotation of the protomers within the AcrB trimer. Drug binding in the protomers occurs mainly at the access pocket (AP) and the deep binding pocket (DBP). Drugs can be sequestered from the periplasm or he outer leaflet of the inner membrane by four different entry channels (CH1–CH4) in the L and T conformations. Professor Pos’s group co-crystallized several antibiotics and dyes in complex with AcrB. Based on these structural insights, extensive mutagenesis and functional analysis of the resulting AcrB variants provided a working hypothesis on the drug specificity of each of the entry channels CH1–CH4, which lead to drug binding in the AP or DBP [13]. For example, high Mw drugs such as ansamycins and macrolides are transported through CH2 and bind initially to the AP in the L protomer, whereas detergents such as β-lactams, and fusidic acid are transported through CH1 and CH4 to the DBP in the T protomer.

To restore activity of antibiotics effluxed by AcrAB-TolC, various research groups have sought to develop AcrB inhibitors. Due to a collaboration with these groups, Professor Pos showed that (i) MBX3132 (Figure 1) inhibits AcrB by binding to the DBP and the hydrophobic trap in the T protomer [14], whereas (ii) BDM88855 (Figure 1) binds to the L protomer in the transmembrane part of AcrB [15].

#### 3.2.2. Pyranopyridine EPIs as Adjunctive Therapies for MDR Enterobacteriaceae, Timothy Opperman, Ph.D. (Microbiotix, Worcester, MA, USA)

Dr. Timothy Opperman is the Director of Microbiology at the anti-infective drug discovery company Microbiotix, Inc. The company is developing a series of novel efflux pump inhibitors that target RND-type pumps in Gram-negative bacteria.

Multidrug-resistant Gram-negative bacteria have become a serious public health issue. In Gram-negative bacteria, intrinsic resistance to antibiotics is based on the (over)expression of multidrug efflux transporters-notably those of the RND family. These efflux pumps are able to extrude and thus inactivate several classes of antibiotics, which explains the pumps’ role in multidrug resistance. A characteristic example is AcrAB/TolC, an efflux pump found in *E. coli* and other Enterobacteriaceae and that can use proton motive force to translocate many classes of antibiotics.

Microbiotix has screened 183,400 compounds (in combination with ciprofloxacin) to find an AcrB inhibitor [16]. After hit identification and optimization, the pyranopyridine MBX-4191 (Figure 1) was chosen as a lead compound. It has no intrinsic antibacterial activity (MIC > 100 µM) and was co-crystallized in the AcrB’s hydrophobic trap; this suggests possible allosteric inhibition [17] or steric hindrance preventing the substrates from binding to AcrB [14].

In vitro, MBX-4191 potentiated various antibiotics, including levofloxacin and tetracyclines. Microbiotix chose to focus its research on improving minocycline’s antibacterial activity. In an in vitro experiment, MBX-4191 lowered minocycline’s MIC90 8-fold in *E. coli* and 16-fold in *K. pneumoniae* and *Enterobacter* spp. The lead compound was also specifically tested in *E. coli* strains possessing a major tetracycline resistance gene tet(B) or tet(D) and in a carbapenem-resistant *K. pneumoniae* strain. Interestingly, MBX-4191 restored minocycline’s antibacterial activity in each of these resistant strains.

After encouraging PK studies in the mouse, MBX-4191 was tested in a murine model of sepsis with a carbapenemase-producing strain of *K. pneumoniae*. The combination of minocycline and the efflux pump inhibitor yielded a dose-dependent reduction in mortality, relative to minocycline alone.

### 3.3. Session 3: Challenging Bacterial Membrane Permeability

#### 3.3.1. Accumulation Rules Lead to Novel Antibiotics for Gram-Negative Bacteria, Paul Hergenrother, Ph.D., Professor (University of Illinois, Urbana-Champaign, IL, USA)

Professor Paul Hergenrother received his B.Sc. in chemistry from the University of Notre Dame in 1994. He went on to the University of Texas at Austin and obtained his Ph.D. in 1999. During this time, Paul was the recipient of an American Chemical Society graduate student fellowship and the Roche Award for Excellence in Organic Chemistry. After an American Cancer Society postdoctoral fellowship at Harvard University, he joined the faculty at the University of Illinois in 2001. His research interests are synthetic organic chemistry, chemical biology, and biochemistry.

Since the discovery of fluoroquinolones in 1968, no new classes of antibiotics against Gram-negative bacteria have emerged. Consequently, the treatment of aggressive Gram-negative infections relies on carbapenems [18]. However, the increase in the prevalence of carbapenem-resistant strains has resulted in more nosocomial infections and a higher mortality rate. There is a critical need for new classes of antibiotics that act against Gram-negative bacteria. This task is complicated by the presence of the double membrane; small molecules have difficulty crossing it and thus accumulating in the cytoplasm [19]. Professor Hergenrother’s group are committed to the discovery of broad-spectrum antibiotics. They have developed a liquid chromatography-tandem mass spectrometry technique for assaying the accumulation of small molecules in Gram-negative bacteria [20]. The results of an initial screening with an in-house compound library showed that positively charged molecules were able to accumulate in *E. coli*. Around 75% of the accumulated compounds contained a primary amine. A further study on primary amine-containing compounds showed that several other parameters were involved in the accumulation process. A total of 297 physical–chemical properties were analyzed in a chemo-informatics screen: globularity and flexibility appeared to be correlated with accumulation. Consequently, the “eNTRy” rules were named to reflect accumulation in *E. coli* when the compound contained an ionizable nitrogen (1° > 2° > 3° amines), had low three-dimensionality (globularity ≤ 0.25), and was rigid (rotatable bonds ≤ 5) [21]. By following these rules, Hergenrother and colleagues were able to convert Gram-positive-only antibiotics into broad-spectrum compounds. One success story is Debio-1452, which is active against S. aureus and against permeability-defective *E. coli*. The compound has low globularity and low flexibility and thus represents a good candidate for conversion into an anti-Gram-negative antibiotic. A primary amine derivative of Debio-1452 (Debio-NH3, Figure 1) was active against wild-type *E. coli* and had enhanced PK properties. Next, around 50 derivatives were synthetized: the resulting Debio-Az-NH3 derivative was the most potent against *E. coli*, *A. baumannii*, and *K. pneumoniae* [22]. Hergenrother and colleagues are continuing to look for novel anti-Gram-negative compounds (i) by defining the accumulation rules for *P. aeruginosa*, which has a higher permeability barrier than other Gram-negative bacteria; and (ii) by extending these rules to other Gram-positive-only compounds. The researchers are also developing definitions of efflux rules, in order to predict the factors that accentuate the active transport of small molecules out of the cell.

#### 3.3.2. Rifabutin for Infusion (BV100) for the Treatment of Severe Carbapenem-Resistant *Acinetobacter baumannii* Infections, Glenn Dale, Ph.D. (BioVersys, Basel, Switzerland)

Dr. Glenn Dale is Chief Development Officer at BioVersys. He is an acknowledged expert in infectious diseases and has authored a large number of publications and patents. Dr. Dale has led BioVersys’ clinical development activities since February 2019, applying his 25 years of R&D experience and significant knowledge of today’s antibiotic development pathways. He obtained his Ph.D. in biochemistry from the University of Basel in 1993. After postdoctoral work in Basel, he successively held the following positions; Group Leader at Roche, Head of Biology and Site Head at Morphochem AG, and Scientific Coordinator with Responsibility for Pre-Clinical Research at Arpida. In 2009, he joined Polyphor, where he led the Antibiotic Research and Early Development team that successfully transitioned murepavadin (POL7080) from the preclinical phase to phase III trials. Glenn is an expert on developing and implementing modern clinical development plans for antibiotic (including pathogen-specific development) and is experienced in presenting to and dialoguing with regulatory authorities in European and the USA (e.g., at scientific advice meetings (MHRA, EMA), type C meetings (FDA), and end-of-phase-II meetings (FDA).

The Gram-negative bacterium *Acinetobacter baumannii* is responsible for 9% of all hospital-treated cases of nosocomial pneumonia [23], for which the mean mortality rate is 53% [24]. These infections are serious because some strains have resistance against most of the weapons in our therapeutic armamentarium (including carbapenems). Accordingly, the WHO has designated carbapenem-resistant *A. baumannii* as a critical priority pathogen for the research and development of new antibiotics. In this context, BioVersys and its collaborators screened the ReFRAME library (a library of 12,000 compounds that have been marketed or are in clinical development) against an extensively drug-resistant (XDR) isolate of *A. baumannii* growing in limited-nutrient RPMI medium (to mimic the in vivo environment) [25]. The spiropiperidyl rifamycin rifabutin (Figure 1) was the most potent hit, with a MIC as low as 0.008 µg/mL. This finding highlighted a novel mechanism of action for rifabutin in *A. baumannii* under nutrient-depleted conditions. It was also found that rifabutin was the only rifamycin able to hijack the TonB-dependent siderophore transporter FhuE and thus enter the bacterium. These in vitro results translated into in vivo efficacy in a mouse model of neutropenic lung infection by an XDR *A. baumannii* strain. Further studies determined that rifabutin’s PK was linear and predictable in neutropenic CD1 mice. As the level of exposure via oral administration was too low to avoid the rapid development of resistance, a new formulation for intravenous (IV) administration was considered [26]. The novel formulation for IV administration developed by BioVersys (BV100) has increased the drug’s solubility 500-fold. BV100 provides greater levels of higher exposure to rifabutin than the oral formulation. Furthermore, BV100′s toxicology profile was similar to that of rifabutin when administered orally in preclinical studies. BV100 is currently being assessed in three phase I studies: NCT04636983, NCT05087069, and NCT05086107.

## 4. Keynote Lectures on Friday, December 10th

### 4.1. Session 1: Stepping Up the Fight against Tuberculosis

#### 4.1.1. First Steps on the Road to TB Drug Candidates: Highlights and Challenges from 10 plus Years of Phenotypic Screening, Robert H. Bates Ph.D. (GSK, Tres Cantos, Spain)

Dr. Robert Bates is Head of Tuberculosis Portfolio at GSK Global Health Pharma R&D. Dr. Bates was trained in organic and medicinal chemistry at the Massachusetts Institute of Technology (BSc) and the Scripps Research institute (Ph.D.). He is now responsible for tuberculosis (TB) research at GSK, with projects ranging from target validation and screening through to early clinical development.

Before the emergence of COVID-19, TB was the infectious pathogen that killed the most people: 1.5 million worldwide in 2020. According to the WHO’s 2021 global TB report, this number increased for the first time in over a decade as a result of the reallocation of human and financial resources from TB to COVID-19 [27,28]. This alarming news reminds us of the urgent need to invest in TB research, and especially in the development of new drugs and regimens for better treatment outcomes in patients with drug-resistant TB. Whereas three decades of TB research have led to an apparently well-filled drug development pipeline (www.newtbdrugs.org, accessed on 17 March 2022), Dr. Bates advocated the discovery and development of many more compounds in order to increase our ability to assemble complementary, synergistic, non-toxic regimens, and alternative treatments that combat drug resistance. Dr. Bates reminded the audience of some of the challenges of target-based screening and showed how phenotypic screening (designed to kill bacteria under conditions determined by the assay) is a key source of leads and drug candidates for treating TB. He also described previous screening programs that balanced in vitro potency early in the discovery process with suitable physical and chemical properties for successful lead optimization. Successful hit selection can also be maximized by the use of in vivo testing as early as possible. Dr. Bates also showed how large screening programs identified interesting new targets, including KasA (to which a target-based strategy was subsequently used to identify novel compounds now in preclinical development).

The development of the preclinical candidate, GSK839 (Figure 1), illustrates some of the strategies adopted by researchers at GSK highlighted by Dr. Bates. It showed that the successful optimization of a compound with moderate efficacy and physical–chemical properties is indeed possible, even before the target had been identified. Dr. Bates then described the use of alternative bacterial growth conditions to identify new compounds and new modes of action, as illustrated by GSK286 (Figure 1), only active in the presence of cholesterol. Over 10 years of intensive screening, Dr. Bates and his collaborators identified several clinical candidates (GSK656, GSK286, and BVL-GSK098, Figure 1) and discovered and validated new druggable targets (such as TrpS and KasA) [29].

#### 4.1.2. Targeting Mycobacterial Phenotypic Variation to Potentiate Therapy and Prevent Persistence, Giulia Manina Ph.D. (Institut Pasteur, Paris, France)

Dr. Giulia Manina heads the Junior Group of Microbial Individuality and Infection at the Institut Pasteur in Paris, France. She was trained in genetics and molecular microbiology at the University of Pavia (Italy), where she worked on the cellular target of a new anti-TB drug. During postdoc research at the École Polytechnique Fédérale de Lausanne (Lausanne, Switzerland), she focused on microfluidic microscopy and the single-cell biology of tuberculosis. Dr. Manina started her own research group in 2015; she is currently building a cutting-edge program on tuberculosis persistence at the single-cell level, using molecular and cell biology tools, microsystems engineering approaches, live cell imaging, and omics. Her group is also involved in tuberculosis drug discovery programs and the identification of subpopulation-based biomarkers.

Double-strand DNA breaks can be lethal for cells if not properly repaired. In mycobacteria experiencing DNA damage, RecA is recruited onto single-strand DNA where it forms a nucleofilament, induces the autoproteolysis of the LexA repressor and activates the SOS-response. Dr. Manina and her colleagues used time-lapse fluorescence microscopy in microfluidic channels to study RecA expression at the single-cell level [30]. They observed a broad range of RecA pulsing levels in the bacterial population-suggesting that level of DNA damage differs from one cell to another. Interestingly, bacilli experiencing high levels of RecA-pulsing were more susceptible to the TB drug ciprofloxacin; this indicates that the drug’s differential efficacy on various bacterial subpopulations is determined by pre-existing variations in DNA damage. Hence, non-pulsing cells are more tolerant of ciprofloxacin killing.

Dr. Manina and her colleagues hypothesized that inducing RecA pulsing would abolish the ciprofloxacin tolerance of the non-pulsing bacterial subpopulation. They therefore designed a screen for identifying compounds that increased RecA pulsing by abolishing cell-to-cell variations and shifting the population towards a homogeneous, ciprofloxacin-sensitive phenotype. This novel single-cell phenotypic assay prompted the technological development of novel, tailor-made microfluidic devices for medium-throughput screening. This strategy identified six hits with additive to synergistic activity when combined with ciprofloxacin, which indicates a partial reversion of drug tolerance. Dr. Manina’s group is currently optimizing the molecules’ efficacy and investigating their mechanism of action. This work was the first to specifically target phenotypic diversity in mycobacteria for drug development in a context of antimicrobial tolerance.

### 4.2. Session 2: New Insights in β-Lactamase Inhibitors

#### 4.2.1. Modulation of the Specificity of Carbapenems and Diazabicyclooctanes for Selective Activity against *Mycobacterium tuberculosis*, Michel Arthur, Ph.D. (Centre de Recherche des Cordeliers, Paris, France)

Dr. Michel Arthur obtained his Ph.D. at the Pasteur Institute in Paris, with work focused on mechanisms of resistance to macrolide antibiotics. As a postdoctoral fellow at Boston University, he worked on bacteriophages and virulence factors in enterobacteria. He then joined the Pasteur Institute as a staff scientist studying the genetics and biochemical mechanisms of resistance to glycopeptide antibiotics in E. faecium. Dr. Arthur extended his expertise in biochemistry through a sabbatical at CNRS-Paris 11, where he initiated a research program on tRNA-dependent aminoacyl transferases (enzymes that catalyze an essential step in peptidoglycan synthesis in Gram-positive bacteria). He was recruited by INSERM as a group leader in 2000 and was promoted as a research unit leader in 2004. Dr. Arthur currently leads a laboratory with around 15 members working on various aspects of cell wall synthesis in relation to antibiotic resistance. His group’s main topics are the design of β-lactams and β-lactamase inhibitors to combat drug resistance, and changes in bacterial cell wall synthesis in response to antibiotic treatments.

As an essential component of bacterial cells, peptidoglycan confers protection against osmotic pressure [31]. The last step in peptidoglycan synthesis involves the formation of peptide crosslinks between adjacent glycan strands and was initially thought to be catalyzed only by penicillin-binding proteins (PBPs). Dr. Arthur’s team discovered a second family of enzymes, the L,D-transpeptidases (LDTs), that also catalyze peptide crosslink formation [32]. LDTs are structurally distinct from PBPs; the former contain a catalytic cysteine instead of a serine and catalyze the formation of different types of crosslinks. The fact that LDT-catalyzed crosslinks account for around 70% of all cross-links in *M. tuberculosis* makes LDTs attractive targets for the development of new antibiotics against tuberculosis [33]. Whereas PBPs are potentially inhibited by all classes of β-lactam, LDTs are only inhibited by carbapenems. The potency of LDT inhibition is determined by the efficacy of acylation of the catalytic cysteine and the stability of the resulting acylenzyme to hydrolysis [34]. Acylation efficacy can be predicted from the β-lactams’ reactivity towards a cysteine residue: the most reactive β-lactams (carbapenems) are the most efficacious LDT inhibitors. Acylation efficacy can be measured by fluorescence kinetics, which also provides information on the structure of the formed covalent adducts [35]. The extent of fluorescence quenching is determined by the status of the β-lactam nitrogen; extensive quenching is observed for intermediates that contain an amine anion and for acylenzymes containing an imine, whereas acylenzymes containing a protonated β-lactam nitrogen give less quenching. Since carbapenems are the most potent LDT inhibitors, three synthetic routes have been developed to modify both of the carbapenem sidechains [36]. The optimized carbapenems were shown to not only inhibit LDTs more avidly but also were less efficaciously hydrolyzed by the *M. tuberculosis* β-lactamase BlaC. LDTs are also inactivated by compounds from the diazabicyclooctane (DBO) family, the first member of which (avibactam) was developed as a β-lactamase inhibitor [37]. Although avibactam shows poor activity against BlaC, it potentiates the antibacterial activity of β-lactams against *M. tuberculosis* through (at least in part) its inhibition of LDTs. Dr. Arthur’s research has shown that β-lactams and DBOs can be optimized to obtain more potent inhibitors of the LDTs needed to build a solid cell wall in *M. tuberculosis*.

#### 4.2.2. Discovery and Preclinical Development of ANT3310, a Broad-Spectrum Serine β-Lactamase Inhibitor Which Potentiates Meropenem against Carbapenem-Resistant Bacteria, David Davies, Ph.D. (Antabio, Labège, France)

Dr. David Davies spent 25 years as a medicinal chemist in the pharmaceutical industry, with GSK. Most of this work was in the antibacterial area: clavulanates, penicillinates, pseudomonates, and bacterial topoisomerase inhibitors. He then worked as a consultant in support of the medicinal chemistry efforts of biotech companies working in the antibacterial area, while simultaneously holding a part-time academic position in the Chemistry Department at University College London; he is still part of the Anderson group. For the past decade, Dr. David Davies has been Head of Medicinal Chemistry at Antabio. He has worked on a variety of antibacterial topics, including beta-lactamase and bacterial elastase inhibitors. Dr. David Davies is named as an inventor on 40 patents and has authored a similar number of papers.

Beta-lactams (the largest important class of antibiotics) act by inhibiting peptidoglycan synthases. The effectiveness of these antibiotics is compromised by the production of β-lactamase enzymes. To circumvent this resistance mechanism, scientists have developed β-lactamase inhibitors capable of inactivating these enzymes and restoring the β-lactams’ activity. The DBOs (a class of serine β-lactamase inhibitors) enhance the β-lactams’ activity against carbapenem-resistant Enterobacterales (CRE) but not against carbapenem-resistant *Acinetobacter baumannii* (CRAB), which are WHO priority pathogens. Ideally, a new DBO antimicrobial should (i) have broad-spectrum activity against all subtypes of serine β-lactamase enzymes, (ii) potentiate the activity of meropenem against resistant Enterobacterales and Acinetobacter strains, and (iii) have a low intrinsic level of antibacterial activity (in order to reduce the potential for resistance). In principle, the introduction of substituents at the 2-position in the DBO should provide novelty while maintaining the compounds’ PK and safety profiles. Hence, electron-withdrawing non-amide substituents were introduced at the 2-position in the DBO [38]. Introduction of a trifluoromethyl group gave a very encouraging level of activity, whereas a CF_2_-2-thiazolyl group decreased the enzyme inhibition. The introduction of a fluorine atom led to the discovery of ANT3310 (Figure 1), which displays a good level of enzyme inhibition and meropenem potentiation. ANT3310 is as effective as avibactam (the first DBO to be approved) against CREs and more active against OXA-23 (the most common variant in CRAB). The X-ray crystal structure of ANT3310 covalently bound to OXA-48 showed that the binding mode was similar to that of avibactam. The ANT3310/meropenem combination was efficacious in mouse models of CRAB and CRE infections. ANT3310 has much the same pharmacokinetic profile as avibactam, and shows good lung retention, low metabolic clearance, low plasma protein binding, a good in vitro safety profile, and no genotoxicity. ANT3310 has received Qualified Infectious Disease Product status from the US FDA for complicated urinary tract infections, complicated intra-abdominal infections, and nosocomial pneumonia. Preclinical development of ANT3310 has been completed, and phase I clinical studies are being prepared.

### 4.3. Session 3: Suffocate the Bug with OXPHOS Inhibitors

#### Quest for Inhibitors of the Mycobacterial Respiratory Terminal Oxidases, Garrett Moraski (Montana State University, Bozeman, MT, USA) and Kevin Pethe, Ph.D., Associate Professor (Nanyang Technological University, Singapore)

Garrett Moraski is a Senior Research Scientist at the Department of Chemistry and Biochemistry, Montana State University. He is also an affiliate of the Department of Chemistry and Biochemistry at the University of Notre Dame. Prior to his academic career, Dr. Moraski worked as a medicinal chemist in industry (for Pfizer, Array Biopharma, and Thios Pharmaceutical) and at a non-profit (SRI International). His research is focused on small-molecule inhibitors of mycobacteria, with particular emphasis on OXPHOS targets.

Dr. Kevin Pethe is an associate Professor and Provost’s Chair in Infectious Diseases at the Lee Kong Chian School of Medicine, Nanyang Technological University (NTU), Singapore. He received his Ph.D. in Genetics and Molecular Biology from the Institut Pasteur de Lille and the University of Lille (France) and pursued his postdoctoral training in cellular microbiology at Cornell University. Dr. Pethe is known for his contributions to chemical biology and antibiotic drug discovery for tuberculosis and mycobacterial diseases. Notably, he led an interdisciplinary team that developed telacebec, a drug candidate for tuberculosis, Buruli ulcer and leprosy that has completed a phase II clinical trial. Before joining NTU, Dr. Pethe gained private sector R&D expertise as a research investigator and project manager at the Novartis Institute for Tropical Disease (Singapore) from 2004 to 2011. In 2011, he joined the Institut Pasteur Korea as a Principal Investigator. In 2013, Dr. Pethe was nominated as Head of the Department of Disease Biology and Chemical Genomics and as acting CEO of Institut Pasteur Korea.

As mentioned above, tuberculosis is still a major global health issue, and new anti-TB compounds are urgently needed. Ideally, these new compounds would have novel mechanisms of action (in order to reduce cross-resistance) and would be effective against persistent subpopulations (in order to shorten the particularly long treatment period for tuberculosis) [39].

In 2004, Garrett Moraski (when at the University of Notre Dame) was part of an anti-TB fragment-screening program based on the design and synthesis of mycobactin analogues; this led to the discovery of oxazole- and oxazoline-based inhibitors of *Mycobacterium tuberculosis* [40]. Exploration of the structure–activity relationships around the oxazole scaffold and the investigation of other heterocycles led to the identification of imidazo[1,2-a]pyridine-based molecules with greater potency [41,42,43]. Even though the compounds’ target had not been clearly identified, Moraski et al.’s transcriptional profiling experiments showed that this chemical series interferes with the bacterium’s energy production by causing upregulation of cytochrome bd oxidase. Development through the Lilly TB Drug Discovery Initiative gave rise to the lead compound ND-10885. Further optimization of this family of compounds is ongoing at Hsiri Pharmaceuticals.

In the meantime, Dr. Pethe and his colleagues at the Novartis Institute for Tropical Diseases found that the proton motive force was essential for the viability of nonreplicating *M. tuberculosis* [44]. They therefore screened chemicals against anaerobic mycobacteria to identify those that targeted ATP homeostasis [45]. Three main chemical series (including imidazopyridines-based molecules) caused the upregulation of cytochrome bd oxidase, as had been observed for the structurally similar analogues developed by Moraski and colleagues. The compounds were not developed further at Novartis, mainly because they were found to mostly be bacteriostatic. After moving to the Institut Pasteur of Korea, Dr. Kevin Pethe led the development of another series of imidazopyridine-based chemical series identified through phenotypic high-content screening in infected macrophages. These analogues were found to target the QcrB sub-unit of the cytochrome bcc complex and thus interfered with oxygen consumption and ATP production. Chemical optimization gave rise to Q203 (telacebec); despite having bacteriostatic properties, this compound is currently in phase II clinical trials [46,47]. The QcrB inhibitors’ bacteriostatic properties can be attributed to cytochrome bd oxidase (the second mycobacterial cytochrome oxidase), which is able to maintain a membrane potential and menaquinol oxidation in the presence of Q203. Indeed, Dr. Kevin Pethe and his colleagues showed that genetic deletion of the cydAB genes encoding cytochrome bd oxidase rendered Q203 bactericidal and were able to clear M. tb infection rapidly in vivo [48]. Hence, Q203 was found to be particularly effective against Mycobacterium ulcerans and Mycobacterium leprae, in which cytochrome bcc is the sole terminal oxidase [49].

With this in mind, Mr Garrett Moraski and Dr. Kevin Pethe joined forces to discover cytochrome bd oxidase inhibitors that would synergize with QcrB inhibitors and thus create a strongly bactericidal combination [50]. Screening in the presence of Q203 led to the identification of a 4-amino-thieno[3,2-d]pyrimidine-based chemical series and a 4-amino-quinazoline-based chemical series [50,51]. The quinazoline ND-11992 is able to boost Q203′s activity in vitro and in vivo. However, this compound displays poor pharmacokinetic properties, and thus, lead optimization is ongoing.

## 5. Workshop

Over the two half-days of the congress, Douglas Häggström from INCATE provided expert advice on research, development, and funding issues in the context of antibiotic drug discovery projects.
